# Crazy People

**Published:** 1860-04

**Authors:** 


### CRAZY PEOPLE.

ONCE upon a sunnier time, when visiting the linen factories of Belfast, a notice was observed above one of the doors, written in Latin, which translated, meant that they would be wise, who, in passing through the world, kept their eyes and ears open. In fact, this makes all the difference between an intelligent man and a know-nothing. Much may any man learn in a large city who will thus employ two of his senses. Lessons of great practical value may be read as well in the narrow alley as on splendid Broadway; in the hovels of the poor as in the magnificent mansions of the Avenue; in the cellars and garrets of the mechanic and the artisan, as well as on change or at the banking-house.

Many an editor and book-writer remembers to have seen in the sixth story of Gray's Mammoth Printing Establishment, a thin, vigilant, snappy-eyed, quick-motioned, sharp-faced gentleman, the cock of the walk in that upper sphere, the lord, ruler, governor, and director of that floor. Always busy, always meeting you with a smile; and yet so picked at and pecked at by Tom, Dick, and Harry, that we have often wondered how he survived. He has been visited regularly for weeks and months by three doctors at least, and he is not dead yet! The man of the Scalpel comes and thunders away, as if he would raise the roof from the building and send it off on a tour of exploration for one of Professor Mitchel's lost stars. Words of wit and wisdom flash out of his mouth, like the scintillations of the great Kohinoor, when they were grinding it into a more comely shape for her majesty, Queen Victoria, long live the same! It is no uncommon thing when Dixon gets on one of his high horses, to have a crowd around him of all the employés, (who are working by the day,) with eyes and mouth open, drinking in the riches of his diction. We have seen it. Then comes Dr. Noyes, his mind ahead of his body; earnest, quick, indefatigable. He, too, gives Powers a twitch or a dig, for the shortcomings of some of Dixon's auditors; and just as that item is settled by the promised rectification of the grievance, in comes the JOURNAL OF HEALTH man, unseen, noiseless, and unknown, and going point-blank at his business in hand, is in the middle of it in double-quick time, every thing being in apple-pie order, and in plain black and white; that is, the black and white are plain enough as to color, although now and then there may be some slight inconvenience in deciding whether the manuscript is French or Mogul; and then pouring in the oil and wine to prepare the way for a welcome next time, he vamooses the ranch, with the sound dying away; "Glad to see you again, Doctor!"

One day we stopped a moment in wonder at the innumerable twitches at Powers. What with the bother of proof-readers and doctors, of calls for more copy, and solicitations of compositors to decipher hieroglyphics; to spell out words that never had any spell in them, or to "make out" words which were of every language under the sun. At length we said to him: "It's a wonder you don't go crazy!"

"Crazy! Haven't time to get crazy; too many things to do," and away he went with a merry laugh, but his words remained, to be a text for some future article in the JOURNAL OF HEALTH.

"Haven't time to get crazy; too many things to do." There is philosophy enough in those dozen words to fill whole tomes of octavos. It's the grand secret of human health and human happiness, to have a plenty to do. "Go ahead, keep moving." There's wisdom in that, and health; health of body, health of brain, and a long life. It's the people who have leisure to mood and mope, and hug sharp-pointed memories, who fill our asylums, and not those who have a dozen irons in the fire at the same time, some round, some square, and some in the pig, so as to bring out and exercise, and develop different mental capabilities, thus making all parts of the brain to grow equally, not only strengthening it but by keeping up equal activities in all its parts, a maturity of judgment and a keenness of discrimination are the result, and these are qualities at the very foundation of human success. The lesson of the article is, as you would avoid an aimless life, a miserable pilgrimage to the land beyond, a mad-house or a premature grave, avoid leisure, avoid one idea, one only pursuit.

